# Living on the edge: Multiscale habitat selection by cheetahs in a human‐wildlife landscape

**DOI:** 10.1002/ece3.4269

**Published:** 2018-07-09

**Authors:** Britt Klaassen, Femke Broekhuis

**Affiliations:** ^1^ Institute of Environmental Sciences (CML) Leiden University Leiden The Netherlands; ^2^ Kenya Wildlife Trust Nairobi Kenya; ^3^ Wildlife Conservation Research Unit Department of Zoology Recanati‐Kaplan Centre University of Oxford Tubney UK

**Keywords:** cheetah, GPS radio‐collars, habitat selection, human‐wildlife landscape, Maasai Mara, multiscale, resource selection functions

## Abstract

Animals select habitats that will ultimately optimize their fitness through access to favorable resources, such as food, mates, and breeding sites. However, access to these resources may be limited by bottom‐up effects, such as availability, and top‐down effects, such as risk avoidance and competition, including that with humans. Competition between wildlife and people over resources, specifically over space, has played a significant role in the worldwide decrease in large carnivores. The goal of this study was to determine the habitat selection of cheetahs (*Acinonyx jubatus*) in a human‐wildlife landscape at multiple spatial scales. Cheetahs are a wide‐ranging, large carnivore, whose significant decline is largely attributed to habitat loss and fragmentation. It is believed that 77% of the global cheetah population ranges outside protected areas, yet little is known about cheetahs’ resource use in areas where they co‐occur with people. The selection, or avoidance, of three anthropogenic variables (human footprint density, distance to main roads and wildlife areas) and five environmental variables (open habitat, semiclosed habitat, edge density, patch density and slope), at multiple spatial scales, was determined by analyzing collar data from six cheetahs. Cheetahs selected variables at different scales; anthropogenic variables were selected at broader scales (720–1440 m) than environmental variables (90–180 m), suggesting that anthropogenic pressures affect habitat selection at a home‐range level, whilst environmental variables influence site‐level habitat selection. Cheetah presence was best explained by human presence, wildlife areas, semiclosed habitat, edge density and slope. Cheetahs showed avoidance for humans and steep slopes and selected for wildlife areas and areas with high proportions of semiclosed habitat and edge density. Understanding a species’ resource requirements, and how these might be affected by humans, is crucial for conservation. Using a multiscale approach, we provide new insights into the habitat selection of a large carnivore living in a human‐wildlife landscape.

## 1 INTRODUCTION

Habitat is a selection of biotic and abiotic factors that provide a space for a species to live (Kearney, 2006). Animals will ideally select habitats that maximize their fitness, whereby longevity and reproduction are increased, by optimizing access to food, mates, and other resources (Orians & Wittenberger, 1991). However, access to favorable resources may be limited by bottom‐up effects, such as resource availability, and top‐down effects, such as risk avoidance and competition. Competition can be between individuals within the same species (intraspecific competition), or between different species needing the same resources (interspecific competition; Kacelnik, Krebs, & Bernstein, 1992; Keddy, 2001). Interspecific competition includes that between wildlife and humans. Numerous studies have shown that human presence can influence species’ distribution and behavior, with the possibility of excluding them from key resources. This has resulted in the decline and range contraction of many mammalian species (Ogutu, Owen‐Smith, Piepho, & Said, 2011; Ripple et al., 2014, 2015). The decline in large carnivore populations and geographic range, for example, are negatively correlated with human densities due to habitat loss and degradation, persecution and depletion of prey (Ripple et al., 2014; Woodroffe, 2000). However, carnivores can reside in human landscapes, be it at lower densities than in wildlife areas as a result of human‐wildlife conflict (Inskip & Zimmermann, 2009). In some cases carnivores might even be attracted to human landscapes because of the availability of domestic and wild prey (Khorozyan, Ghoddousi, Soofi, & Waltert, 2015; Linnell et al., 2005) and anthropogenic food sources (Cozzi et al., 2016), or because it acts as a refuge from competitors (van der Meer, Fritz, Blinston, & Rasmussen, 2014). As the global human population continues to increase, it is crucial to understand if, and how, carnivores and people can coexist (Carter & Linnell, 2016; Oriol‐Cotterill, Macdonald, Valeix, Ekwanga, & Frank, 2015). One approach is by determining the anthropogenic and environmental drivers that influence the habitat selection of carnivores in landscapes where they co‐occur with people.

Habitat selection studies often use a single scale approach, however, there is increasing evidence that biological, ecological, and geographical processes occur at different spatial scales (Cushman & Huettmann, 2010). For example, Timm, McGarigal, Cushman, and Ganey (2016) assessed a multiscale habitat selection for nesting and roosting areas of Mexican spotted owl (*Strix occidentalis lucida*) and compared this to a single scale habitat selection. They found that the multiscale habitat selection model outperformed the single scale model. Individuals may first select their area of use, i.e. home‐range, which is followed by selection for different resources, such as food, within this home‐range. In other words, factors important for home‐range selection may be selected on a broad scale, whereas resources may be selected on a fine scale (Boyce, 2018). Thus, taking multiple scales into consideration is necessary in order to accurately describe species–habitat relationships (Cushman & McGarigal, 2004), yet multiscale habitat selection studies of terrestrial carnivores are still uncommon (but see Cushman, Elliot, Macdonald, & Loveridge, 2016; Elliot, Cushman, Macdonald, & Loveridge, 2014).

Here we investigate the multiscale habitat selection of cheetahs (*Acinonyx jubatus*) in a landscape where they co‐occur with people. Cheetahs are a wide‐ranging, large carnivore whose significant population decline has largely been attributed to habitat loss and fragmentation. As a result, cheetahs have disappeared from 91% of their historic range with a current population standing at only ~7,100 wild individuals (Durant et al., 2017). For a cheetah population to be viable it needs a contiguous area of approximately 4,000–8,000 km^2^ of suitable habitat (Durant, Bashir, Maddox, & Laurenson, 2007), yet very few protected areas in Africa are larger than 4,000 km^2^ (Durant et al., 2017). As a result, 77% of the global cheetah population is believed to range outside protected areas (Durant et al., 2017). Despite this, cheetahs’ resource selection within landscapes where they co‐occur with people is still poorly understood. This is mainly because previous habitat selection studies have been conducted in fenced wildlife areas or in areas where the human population density is low, and therefore encounters with people, other than tourists, are minimal (e.g., Bissett & Bernard, 2007; Broomhall, Mills, & Toit, 2003; Broekhuis, Cozzi, Valeix, McNutt, & Macdonald, 2013; Pettorelli, Hilborn, Broekhuis, & Durant, 2009; Welch, Bissett, Perry, & Parker, 2015). These studies have however found that certain environmental factors, such as vegetation and habitat structure, influence cheetahs’ fitness, as it can affect hunting success (Mills, Broomhall, & Du Toit, 2004), cub survival (Broekhuis, 2018) and coexistence with other predators (Broekhuis et al., 2013).

**Figure 1:**
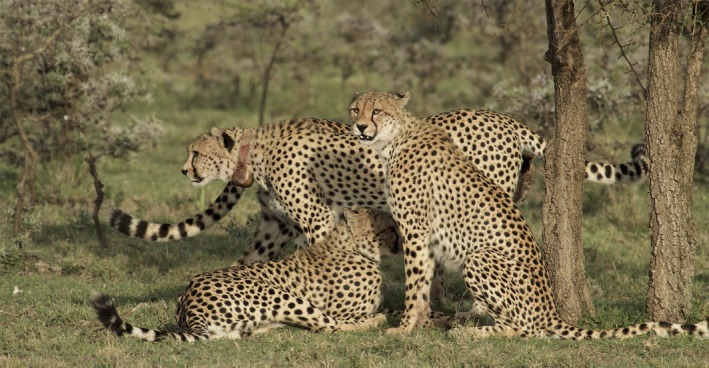
Cheetah (M01), part of a five‐male coalition, with a GPS radio‐collar in the Maasai Mara, Kenya

The Maasai Mara in Kenya is an ideal place to conduct this study as it is a landscape that is under increasing human pressure (Lamprey & Reid, 2004) and yet it boasts a high density of cheetahs (Broekhuis & Gopalaswamy, 2016). The area consists of both wildlife areas (dominated by wildlife) and community land (dominated by people), with no barriers separating the two so that cheetahs and other animals can move freely. We will therefore refer to the landscape as a human‐wildlife landscape rather than a human‐dominated landscape. The Maasai Mara also consists of a mosaic of open and wooded (semiclosed) habitat types (Oindo, Skidmore, & De Salvo, 2003). Here we investigate the influence of anthropogenic and environmental factors, at different scales, on the habitat use of cheetahs residing in a human‐wildlife landscape using data from six cheetahs fitted with GPS radio‐collars (Figure 1). Various studies on carnivores in human‐dominated landscapes have shown a strong avoidance of humans (e.g., Elliot et al., 2014), however, it is possible that cheetahs may prefer human‐dominated areas as it is believed that they do well in areas where competitors, particularly lions (*Panthera leo*) and spotted hyaenas (*Crocuta crocuta*), have been eradicated (Marker, Dickman, Mills, & Macdonald, 2003). Under a competition‐avoidance hypothesis we would expect cheetahs in the Maasai Mara to prefer areas outside the wildlife areas as the densities of competitors, especially lions, are high inside the wildlife areas (Elliot & Gopalaswamy, 2017). Alternatively, if the human disturbance outside the wildlife areas is high, then cheetahs are likely to select for wildlife areas. We would also expect that cheetah habitat selection will be influenced by factors that provide concealment from other predators, including humans, such as semiclosed habitat, and those that provide opportunities for increased hunting success, such as areas with a high edge density between open and semiclosed areas and areas with a gentle slope. Lastly, we expect that the anthropogenic and environmental factors are selected at different scales, with factors important for cheetahs’ choice in area utilization selected at a broad scale and resources within home‐ranges to be selected at a fine scale.

## 2 MATERIAL AND METHODS

### 2.1 Study area

**Figure 2:**
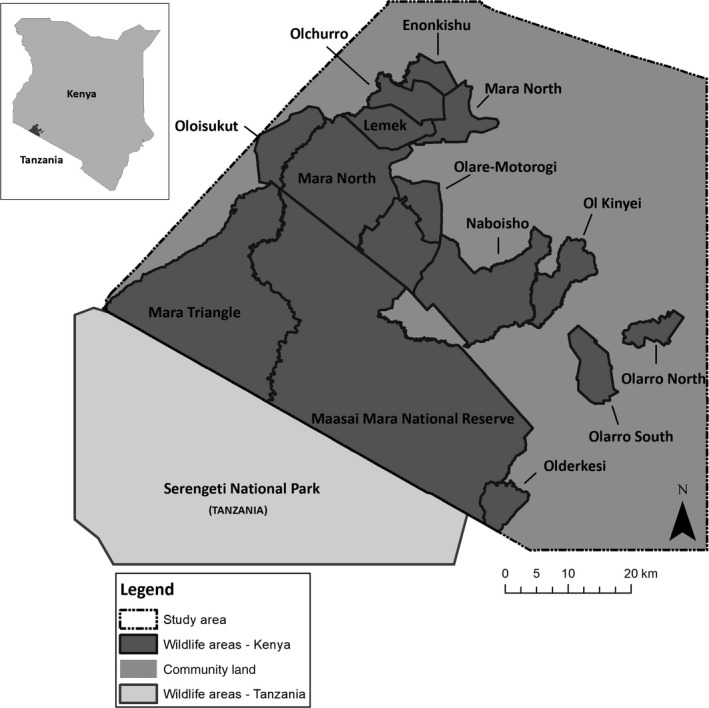
Map of the study area in the southwest of Kenya

This study was conducted in the Maasai Mara which lies in the southwest of Kenya (centered at 1°S and 35°E) and it makes up the northern section of the larger Serengeti‐Mara ecosystem. The study area itself (~5,762 km^2^) included both wildlife and non‐wildlife areas (community land; Figure 2). The wildlife areas (~2,601 km^2^) are set aside for wildlife‐based activities, such as photographic tourism, and include the Maasai Mara National Reserve (MMNR) and the surrounding conservancies. The MMNR is managed by the Narok County Government while the conservancies are each managed by different management companies. The conservancies are formed through a partnership between Maasai landowners and tourism companies, whereby landowners receive a fixed, monthly payment for leasing their land for wildlife based activities on the condition that they do not live on the land, cultivate or develop it (Osano et al., 2013; Thompson, Serneels, Kaelo, & Trench, 2009). However, in some cases, especially on the boundaries of some conservancies, people still reside with their livestock. The wildlife areas are not fenced making it possible for animals to move freely into community land.

Community land (~3,161 km^2^) is the area outside the MMNR and the conservancies, of which the north and west are dominated by agriculture. To the east settlements are predominant, where the Maasai people reside with their livestock in homesteads known as *manyattas*. Both people and livestock in the area are increasing at a rapid rate (Lamprey & Reid, 2004), as are the fences that are being erected as a result of land subdivision (Løvschal et al., 2017).

The habitat in the study area varies greatly, ranging from open grasslands and shrubland, to riverine forests (Oindo et al., 2003). The open grassland plains, which are dominated by *Themeda triandra*, are mostly found toward the south and west of the study area, while the north and northeast consist mostly of Croton thickets (*Croton dichogamous*) and Vachellia woodlands (*Vachellia drepanolobium* and *V. gerrardii*). Riverine woodland can be found along the major rivers and their tributaries (Oindo et al., 2003).

The area experiences one rainy season (November–June) and one dry season (July–October; Ogutu, Piepho, Dublin, Bhola, & Reid, 2008). The long grass after the rainy season attracts large numbers of migratory ungulates, including the white‐bearded wildebeest (*Connochaetes taurinus*) and the common zebra (*Equus quagga*) from the Serengeti in Tanzania. Throughout the year, a large abundance of cheetah prey is still available, including resident white‐bearded wildebeest, Thomson's gazelle (*Eudorcas thomsonii*), Grant's gazelle (*Nanger granti*) and impala (*Aepyceros melampus*; Broekhuis, Thuo, & Hayward, 2018).

### 2.2 Cheetah collaring

Global Positioning System (GPS) radio‐collars (African Wildlife Tracking—http://www.awt.co.za) were fitted on six adult cheetahs (four males and two females) between 11 April 2015 and 16 August 2017. Each of the collared individuals were singletons, except for one male (M01) who was part of a five‐male coalition and one female (F02) who had four 14‐month‐old cubs at the time of capture. While this is a relatively small sample size, it represents ~30% of the adult population as it is estimated that only 32 individuals >18 months are found within the wildlife areas of the Maasai Mara (Broekhuis & Gopalaswamy, 2016).

In compliance with Kenyan law, all immobilizations for deployment/removal of radio‐collars were performed by a Kenya Wildlife Service veterinarian. Cheetahs were free‐darted and immobilized using a combination of ketamine (2–2.5 mg/kg) and medetomidine (0.07 mg/kg), remotely administered by a Dan‐Inject CO_2_ rifle (Dan‐Inject, Denmark), and reversed with atipamezole (0.3 mg/ml; following Kock, Meltzer, & Burroughs, 2006). Sedation time was kept to a minimum, typically less than 1 hr. After immobilization, all cheetahs recovered fully, showing no signs of distress and no apparent side effects were observed on both the short‐ and long‐term. Collars were only fitted on adults and weighed 400 g which is the recommended weight for cheetah collars (Broekhuis, Bissett, & Chelysheva, 2017a). All radio‐collars were removed if they malfunctioned or if the batteries were low.

Collars deployed on females collected GPS coordinates every 2 hr (01, 03, 05, 07, 09, 11, 13, 15, 17, 19, 21, 23 hr), but, due to problems with battery life, the collars on males were set to collect GPS coordinates every 3 hr (00, 03, 06, 09, 12, 15, 18, 21 hr). On average, collars were deployed for 156 days, ranging from 60 to 301 days (Supporting Information Table S1).

### 2.3 Habitat selection

Habitat selection was determined using a resource selection function (RSF), whereby we compared what was used by the cheetahs, using the location data collected by the collars, to what was available to them within the study area (Manly, McDonald, Thomas, McDonald, & Erickson, 2002). To determine use, we randomly selected 50% of the total points collected at night and 50% of the total points collected during the day per individual to minimize autocorrelation. One female (F01) had a litter after her collar was deployed, which she lost after 22 days. As her movements were restricted during this time, all data points, except for one randomly selected point per day during this period, were removed. To determine the number of available points that were needed for the analysis, we conducted a sensitivity analysis following the methods described by Stabach, Wittemyer, Boone, Reid, and Worden (2016). Based on this we decided to use a 1:1 ratio for used and available points, meaning that we created the same number of random points as GPS points. The random points were generated within the 99% utilization distribution of all the individuals combined. This utilization distribution was calculated using the Kernel Density Estimation function in Geospatial Modeling Environment (Beyer, 2012) using a Gaussian distribution, as this is representative of cheetah movement (Broekhuis & Gopalaswamy, 2016), and least squares cross‐validation method to estimate the optimized kernel bandwidth matrix. Habitat selection was assessed both during the night as well as during the day (night: 7 p.m.–6 a.m.; day: 7 a.m.–6 p.m.).

### 2.4 Explanatory variables

We included three anthropogenic and five environmental explanatory variables in our analyses, which we grouped into five classes, based on their similarities: anthropogenic pressure, wildlife areas, habitat type, habitat structure, and slope.

#### 2.4.1 Anthropogenic variables

##### Anthropogenic pressure

Two variables were used to quantify the anthropogenic pressure within the study area: the human footprint density and the distance to main roads. The human footprint, which included human development, such as settlements, livestock enclosures, dams, towns and agricultural land, was digitized using QGIS (QGIS Development Team, 2017) with the OpenLayers plugin for both Google Earth and Bing maps. To reflect the size of the development, polygons were drawn around each human development. This was combined with the polygons of fences within the study area based on 2015 and 2016 fence data from Løvschal et al. (2017). The polygons were converted to points and using the *point density* function in ArcGIS 10.2.2 (Environmental Systems Research Institute Inc., 2014) the density of human footprint was calculated for the different scales (see “Scaling”). The combination of the human footprint and the 2015 fence data were used for cheetahs collared in 2015 and the combination of the human footprint and the 2016 fence data were used for cheetahs collared in 2016 and 2017. For the distance to main roads, the Euclidean distance to roads was calculated using the Spatial Analyst tool in ArcGIS.

##### Wildlife areas

This included the MMNR and the conservancies.

#### 2.4.2 Environmental variables

##### Habitat type

We created a habitat map based on two LandSat 8 images (17 July 2013 and 25 January 2014). The images were classified based on habitat structure using the Random Forest method, chosen for its high classification accuracy (Cutler et al., 2007; Kampichler, Wieland, Calmé, Weissenberger, & Arriaga‐Weiss, 2010). The training data were created in QGIS using a combination of 378 habitat points obtained on the ground and high‐resolution SPOT 5 imagery (2.5 m resolution) from 2011 (SPOT data/ISIS programme, Copyright CNES). In addition to the original satellite images, we also used the Normalized Difference Vegetation Index and texture to increase the accuracy of classification. The classification was carried out using the *randomForest* package in R (R Core Team, 2017). Habitat was classified according to three different habitat types: open, semiclosed and closed (Supporting Information Table S2). The final map was ground‐truthed based on 2,000 random points and had an accuracy of 87%. For this analysis, we used the two most dominant habitat variables within the study area: open and semiclosed habitat (Supporting Information Table S3).

##### Habitat structure

Two variables, edge density and the patch density, were used as these variables have been shown to be important habitat structures for cheetahs (Mills et al., 2004). Both variables were calculated in FRAGSTATS, version 4.2.1.603 (McGarigal & Ene, 2015) using the habitat map (see above). Edge density represents the total edge length between open and semiclosed habitats divided by the total landscape area in squared meters which is then converted into hectares. This results in a standardized edge density, which can be compared along different sized landscapes. Patch density represents the number of open and semiclosed patches per 100 hectares. Patch density is calculated by dividing the number of patches of each habitat type by the total number of patches, converted to 100 hectares, so that landscapes of different sizes can be compared.

##### Slope

The slope was calculated using the function *Slope* in the Spatial Analyst toolbox in ArcGIS. We used digital elevation data for this calculation, which was downloaded from https://earthexplorer.usgs.gov using the Shuttle Radar Topography Mission 1 Arc‐Second Global dataset.

All the predictor variables, except those that were proportions, were standardized using a *z*‐score transformation with a mean of 0 and a standard deviation of 1.

#### 2.4.3 Scaling

All the variables were based on a spatial resolution of 30 × 30 m. Each of the environmental and anthropogenic variables, apart from the slope and distance to main roads, were calculated at six different scales: 90, 180, 360, 720, 1,440, and 2,880 m to determine at which scale selection was biologically meaningful (McGarigal, Wan, Zeller, Timm, & Cushman, 2016). These scales were chosen as we wanted to include a wide variance of scales, but were limited by our original 30 × 30 m resolution. The different scales were computed using the program FRAGSTATS, using moving window statistics (Isaaks & Srivastava, 1989). To create the different scales of the categorical variables (open habitat, semiclosed habitat, and wildlife areas) the proportion of each category was calculated using the Percentage of Landscape (PLAND) option for class metrics, whereas the different scales of the edge and patch densities were calculated as a landscape metric.

### 2.5 Statistical analyses

Cheetah habitat use was determined using Generalized Linear Mixed Models (GLMMs) with a binomial error structure with 1 representing the actual GPS locations of the cheetahs (used) and 0 representing the random points (available). Cheetah ID was included as a random factor to account for differences between individual cheetahs (Gillies et al., 2006). For each analysis, the Akaike Information Criterion (AIC) was used to select the best model, with the lowest AIC‐value representing the best model (Burnham & Anderson, 2002). All our statistical analyses were performed in R (R Core Team, 2017).

Our analysis followed a multi‐stage process. First, for each explanatory variable, a univariate scaling analysis was performed to optimize the scale that best captured the cheetahs’ response (McGarigal et al., 2016). The scale with the lowest AIC value was then retained for the next step. Second, using the explanatory variables with the scales from the previous step, the most important variable in each of the classes was selected and used for the final GLMMs in order to avoid overspecification of the models. Lastly, we conducted an all‐subsets analysis using the most important variable from each of the five classes. Variance inflation for the five variables was checked using the *vif* function in the package *car*. The different models were ranked using AIC and relative support was assessed using Akaike weights (*w*_*i*_). When one model was superior (*w*_*i*_ **> 0.9) this was used, otherwise parameter estimates were averaged for models with AIC differences (Δ_*i*_ **< 2) correcting for model weights (Burnham & Anderson, 2002).

The parameters of the best model were used to create the habitat suitability map using the resource selection function by the exponential form of

$$w\left(x\right)=\exp\left(\beta_{0}+\beta_{1}x_{1ij}+\beta_{2}x_{2ij}+\cdots +\beta_{n}x_{\mathrm{nij}}+\gamma_{0j}\right)$$

whereby *w*(*x*) is the outcome of the RSF, *x*_*n*_ the covariates, *β*_*n*_ the fixed regression coefficients for locations *i* and individuals *j*,* β*_0_ the mean intercept and *γ*_0*j*_ the random intercept for individuals *j* (Manly et al., 2002; Gillies et al., 2006).

## 3 RESULTS

**Figure 3:**
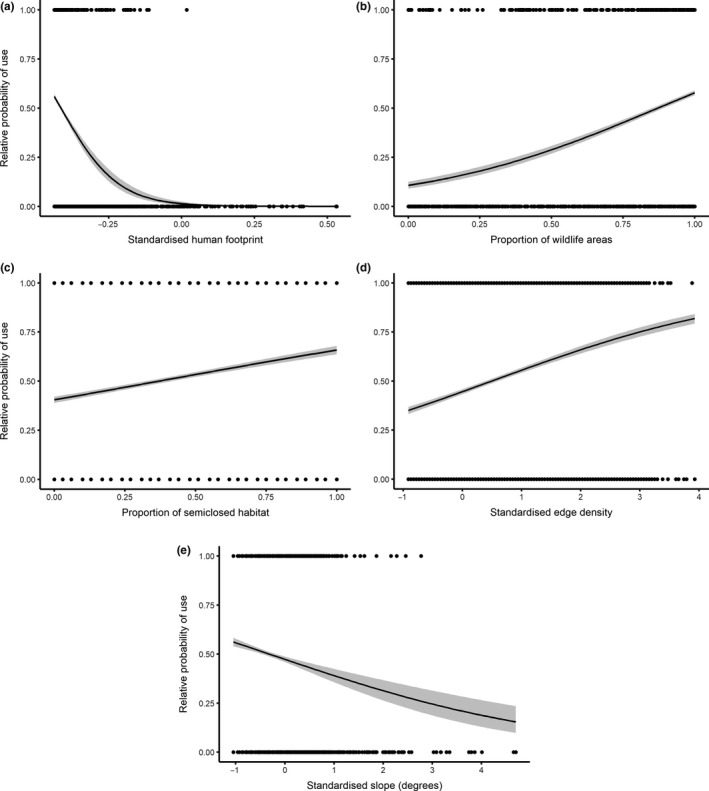
The relative probability of use by cheetahs in the Maasai Mara in relation to (a) human footprint density, (b) wildlife areas, (c) semiclosed habitat, (d) edge density, and (e) slope. The fitted lines are presented with the 95% confidence intervals in grey

**Figure 4:**
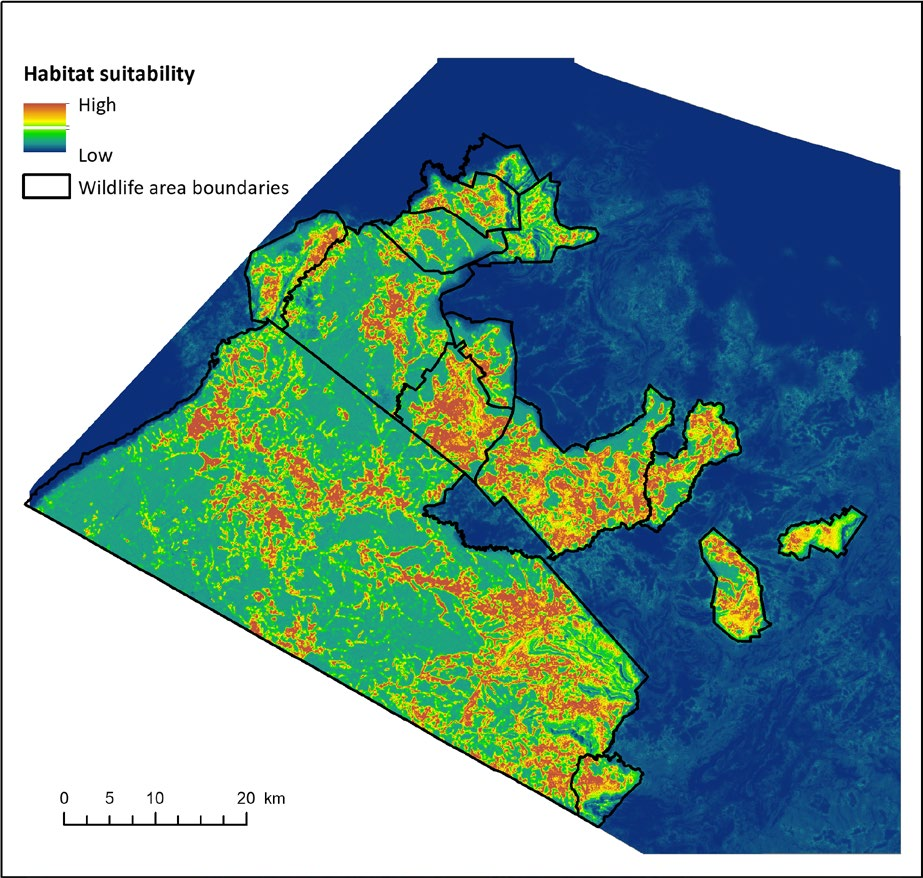
Habitat suitability map for cheetahs in the Maasai Mara, Kenya, based on the following variables: human footprint density, wildlife areas, semiclosed habitat, edge density and slope, with the wildlife areas outlined in black. The map was drawn using the Stretch type “Standard Deviations” in ArcGIS

Cheetah habitat selection was similar during the day and night, hence we decided to pool the data (Supporting information Figure S1). The univariate analysis shows that the explanatory variables influenced the distribution of cheetahs on different scales. Most notably, the human footprint density and the proportion of wildlife areas influenced cheetah habitat selection at a scale of 1,440 and 720 m, respectively, whereas the environmental variables had a much finer sphere of influence ranging between 90 and 180 m (open habitat: 90 m; semiclosed habitat: 90 m; edge density: 180 m; and patch density: 90 m). Human presence was a better indicator of cheetah presence than distance to main roads (Supporting Information Table S4). Within the habitat type and structure, cheetah distribution was better explained by semiclosed habitat rather than open habitat and edge density rather than patch density (Supporting Information Table S4). This meant that the variables that were used in the final analysis included the human footprint density, wildlife areas, semiclosed habitat, edge density, and slope. All of these variables were included in the top model (Table 1), but their effect on habitat use varied per variable with those with negative coefficients being avoided and positive coefficients being selected. Most notably was the avoidance of humans (estimate = −2.725, 95% CI = −4.162 to −1.420; Figure 3a) and selection for wildlife areas (estimate = 2.358, 95% CI = 2.122 to 2.601; Figure 3b). Cheetahs also selected for areas dominated by semiclosed habitat (estimate = 1.089, 95% CI = 0.937 to 1.242; Figure 3c) and areas where there was a high edge density between open and semiclosed habitat (estimate = 0.334, 95% CI = 0.282 to 0.386 Figure 3d), but avoided areas with steep slopes (estimate = −0.260, 95% CI = −0.380 to −0.141; Figure 3e). These results were used to create a habitat suitability map for the study area (Figure 4).

**Table 1:** Top ten GLMMs representing cheetah habitat selection in the Maasai Mara, Kenya

Model structure	AIC‐value	Δ_*i*_	*w*_*i*_
hf_1440_ + wa_720_ + sch_90_ + ed_180_ + sl	8636.2	0.00	1
hf_1440_ + wa_720_ + sch_90_ + ed_180_	8652.7	16.48	0
wa_720_ + sch_90_ + ed_180_ + sl	8653.0	16.75	0
wa_720_ + sch_90_ + ed_180_	8667.5	31.22	0
hf_1440_ + wa_720_ + sch_90_ + sl	8797.5	161.27	0
wa_720_ + sch_90_ + sl	8814.2	177.92	0
hf_1440_ + ed_180_ + wa_720_ + sl	8828.8	192.60	0
hf_1440_ + sch_90_ + wa_720_	8836.5	200.21	0
ed_180_ + wa_720_ + hf_1440_	8842.0	205.71	0
sch_90_ + wa_720_	8842.0	206.10	0

*Note*.

The most parsimonious model was found to be the most complex model, with hf = human footprint density, wa = wildlife areas, sch = semi‐closed habitat, ed = edge density, sl = slope, and the appropriate scales as subscripts. Δ_*i*_ represents the AIC difference and *w*_*i*_ represents the model weightings.

The habitat suitability map shows that the wildlife areas are more suitable for cheetahs than community land. Within the wildlife areas, the northern tip had the least suitable habitat for cheetahs, whereas in the eastern areas (Olare‐Motorogi Conservancy, Naboisho Conservancy, Ol Kinyei Conservancy and the eastern section of the MMNR) were the most suitable. Even though the community land shows poor habitat suitability, a similar pattern can be seen. Most of the northern section indicate very poor cheetah habitat (bright‐blue), while the eastern section shows to be slightly better (light‐blue), possibly providing connectivity between nonadjacent conservancies when protected.

## 4 DISCUSSION

This study explored multiscale habitat selection by cheetahs in a human‐wildlife landscape. Our results reveal that anthropogenic variables were selected at broader scales than environmental variables. The best indicators of cheetah presence were the human footprint density, wildlife areas, semiclosed habitat, edge density and slope. Cheetahs selected for areas with a low human footprint density and a high proportion of wildlife areas, semiclosed habitat, and edge density but avoided areas with steep slopes.

### 4.1 Scales

Cheetahs selected anthropogenic variables, including human footprint density (1,440 m) and wildlife areas (720 m), at a broader scale compared to the environmental variables (90 and 180 m). This suggests that anthropogenic pressures affect habitat selection at a home‐range level, whilst environmental variables influence site‐level habitat selection (Boyce, 2018; DeCesare et al., 2012). A similar result was found for brown bears (*Ursus arctos*) in the Cantabrian Range, northwest Spain (Sánchez, Cushman, & Saura, 2014). Brown bears within this region perceived anthropogenic disturbances, such as building density, agriculture, and transportation infrastructure, at broad scales, while environmental variables, such as edge effects amongst cover types and canopy closure, were selected at finer scales (Sánchez et al., 2014). Here, we show the importance of taking scale into account when conducting habitat selection studies, which is supported by other studies on, for example, Mexican spotted owl (Timm et al., 2016), mountain bongo antelope (*Tragelaphus euryceros isaaci*; Estes, Okin, Mwangi, & Shugart, 2008) and brown bears (Sánchez et al., 2014). However, the multiscale approach within habitat selection studies is still uncommon (McGarigal et al., 2016) and to our knowledge this is the first multiscale habitat selection study for cheetahs.

### 4.2 Anthropogenic variables

Under the competition‐avoidance hypothesis we expected that cheetahs would prefer areas outside the wildlife areas to avoid competition with other predators, especially lions as they occur at very high densities inside the wildlife areas (Elliot & Gopalaswamy, 2017). However, our results show a strong preference for wildlife areas and an avoidance of human presence. This is in line with findings by Riggio et al. (2018) who classified cheetahs as being very sensitive to human disturbances based on a combination of transect data and interview data in Wami‐Mbiki Wildlife Management Area, Tanzania. Marker et al. (2003), on the other hand, argue that cheetahs in Namibia prefer the farmlands over wildlife areas to avoid lions and spotted hyaenas. It is possible that the human density outside wildlife areas in the Maasai Mara is higher compared to the farmlands in Namibia (Lamprey & Reid, 2004; Marker, Dickman, Mills, Jeo, & Macdonald, 2008). These results therefore suggest that cheetahs potentially consider humans as a bigger threat than other predators. This is similar to findings by Clinchy et al. (2016), who found that mesopredators were more fearful of humans than larger predators.

What was striking about the data and the subsequent results was that not only did cheetahs prefer the wildlife areas, they rarely left the wildlife areas despite frequently coming close to the boundaries of the wildlife areas (Supporting Information Figure S2). None of the wildlife areas are fenced making it possible for cheetahs to move into the more human‐dominated areas. However, a high number of human settlements are found on the borders of the wildlife areas, possibly creating a barrier. This barrier is not a hard boundary as there is occasional movement of cheetahs in and out of the wildlife areas. Only one cheetah within our study (F02) frequently traveled through community land between two nonadjacent conservancies (Supporting Information Figure S2). This finding is corroborated by a recent study by Madsen and Broekhuis (in press) who found that cheetahs are occasionally seen outside the wildlife areas, but that cheetahs were more likely to occur close to the wildlife areas. A big concern for carnivores moving through, or residing in, human‐dominated areas is the potential for conflict to occur with humans. In the Maasai Mara, cheetahs do occasionally predate on livestock (Broekhuis, Thuo, & Hayward 2018) and as livestock numbers outside the wildlife areas are high (Broekhuis, Cushman, & Elliot, 2017b; Ogutu et al., 2016) it is potentially an easy food source for cheetahs. However, it has been shown that some carnivores, including cheetahs, prefer wild prey over domestic (Ghoddousi et al., 2016; Marker, 2002) and as wild prey species outside the wildlife areas are declining (Ogutu et al., 2016) it is possible that cheetahs prefer the wildlife areas where large numbers of wild prey are still readily available.

When including anthropogenic variables in this habitat selection study two assumptions were made. Firstly, we did not differentiate between the different wildlife areas, and thereby assumed that all the wildlife areas had the same management policies. This is, however, not the case as management policies varied greatly between the different wildlife areas (Bedelian & Ogutu, 2017). Most notable is that each wildlife area has a different policy on livestock grazing; in some areas livestock grazing within the wildlife areas is prohibited whereas in other areas livestock grazing is allowed either all year round or in designated areas or during specific times of year. There is little research done on the influence of the presence of livestock and herders on carnivore behavior, but considering the strong avoidance of human presence by cheetahs it is possible that they minimize the use of wildlife areas where large herds of livestock are allowed to graze continuously.

Secondly, the human footprint density layer was created using Google Earth and Bing maps with some images dating back to 2009. As the human population is continually increasing (Lamprey & Reid, 2004) it is likely that the human footprint data used in this study was an underestimation of the current human pressures that cheetahs face. However, as cheetahs showed an avoidance of humans, which is corroborated by other studies (e.g., Riggio et al., 2018), we suspect that our results are on the conservative side.

### 4.3 Environmental variables

The global cheetah decline has, in some part, been attributed to predation and competition with other predators, especially lions and spotted hyaenas. Laurenson (1995) estimated that in Serengeti National Park, Tanzania only 4.8% of cubs born reach independence, with 73% of deaths accounted for by predator‐induced mortality. However, studies in other parts of Africa have found a much higher cub survival, even with the presence of lions (e.g., Mills & Mills, 2013). Additionally, recent research has shown that lion numbers do not negatively influence cheetah numbers (Swanson et al., 2014), which is likely because cheetahs adjust their spatiotemporal patterns on a fine scale to avoid immediate risks of these larger, more dominant carnivores (Broekhuis et al., 2013; Vanak et al., 2013). We found that cheetahs preferred areas dominated by semiclosed habitat which could explain why cheetahs preferred the wildlife areas despite the very high lion densities (Elliot & Gopalaswamy, 2017). Semiclosed habitat provides concealment, thereby minimizing the possibility of being detected by other predators. This is supported by findings from the Okavango Delta, Botswana, where cheetahs were able to use the same areas as lions especially in more wooded habitat (Broekhuis et al., 2013). The use of semiclosed habitat could similarly be a key in avoiding humans. Both Eurasian lynx (*Lynx lynx*) and coyotes (*Canis latrans*) showed a preference for dense vegetation in areas with high human disturbance (Atwood, Weeks, & Gehring, 2004; Bouyer et al., 2015). This was primarily linked to the fact that dense vegetation can provide cover and thus security. By selecting areas that provide more coverage, an individual may increase its fitness by increasing its longevity. This illustrates the importance of conserving dense vegetation patches both inside and outside wildlife areas.

Additionally to selecting areas dominated by semiclosed habitat, cheetahs also selected for areas with a high density of edges between open and semiclosed habitat. Previous studies on cheetahs in the Serengeti National Park in Tanzania suggest that cheetahs’ high speed hunting strategy requires them to primarily use open plains (Caro, 1994). However, other studies have shown that cheetahs can increase their hunting success using a combination of woody vegetation and open plains (Bissett & Bernard, 2007; Mills et al., 2004), using wooded areas to stalk and the open habitat to pursue and catch prey (Mills et al., 2004). Similarly, cougars (*Puma concolor*) made significantly more kills in edge and edge like areas (areas with trees with a visibility of at least 20 m) as these areas provided an opportunity for cougars to detect prey, while staying hidden during the stalking phase of the hunt (Laundré & Hernández, 2003).

Cheetahs within this study selected against slopes which is in contrast to findings by Welch et al. (2015). Welch et al. (2015) found that cheetahs selected for steep slopes in Mountain Zebra National Park, South Africa however, they found significant variation between individuals. Some individuals, especially males and those that were introduced at a later date, selected for less steep slopes within this area. Furthermore, it is possible that our findings are different to those by Welch et al. (2015), because slopes in the Maasai Mara are steeper (maximum slope = 44.45°) than those found in Mountain Zebra National Park. Studies on other carnivores in human‐dominated landscapes, including brown bear and Eurasian lynx, have shown that steep slopes are preferred as it provides a refuge from human disturbances (Basille, Calenge, Marboutin, Andersen, & Gaillard, 2008; Bouyer et al., 2015; Petram, Knauer, & Kaczensky, 2004). However, we found that cheetahs avoided steep slopes probably as it limits their hunting ability within our study area.

Numerous studies on habitat selection of carnivores have shown the importance of taking prey densities into account (e.g., Basille et al., 2009; Durant, 1998). However, it was not possible to include this environmental factor within our study. Prey densities within the Maasai Mara are very high (Stelfox, Peden, Epp, Hudson, & Susan, 1986), but fluctuate tremendously on a daily basis and throughout the year (Bhola, Ogutu, Said, Piepho, & Olff, 2012), making it difficult to accurately acquire numbers that could be used for a habitat selection study. However, as prey is not scarce within this study area, and cheetahs’ ability to avoid immediate risks from more dominant carnivores attracted to higher prey numbers, we suspect that excluding this factor from our analysis has not significantly impacted our results.

## 5 CONCLUSION AND RECOMMENDATIONS

Overall, our results indicate a strong avoidance of cheetahs to human pressures, proving the importance of areas that are set aside for wildlife. This study also shows the importance of taking different scales into account when investigating habitat selection. While cheetahs were found to prefer wildlife areas, there is possible suitable habitat outside the wildlife areas which would be important for connectivity. However, to better predict corridors, step and path selection functions are more useful, as opposed to the more traditional point selection function that we have used (Zeller, McGarigal, & Whiteley, 2012). Additionally, our study did not include selection of habitat for different behavioral or demographic states. For example, Elliot et al. (2014) looked at patterns of connectivity for lions, and found a substantial difference between females, males, and dispersing males. Similarly, Abrahms et al. (2016) found that African wild dogs (*Lycaon pictus*) use roads differently depending on their behavioral state. Wild dogs selected roads when travelling, specifically in more dense vegetation, while they ignored roads when running at high speeds and avoided roads all together when resting (Abrahms et al., 2016). In order to accurately identify corridors within the Maasai Mara, we strongly recommend further research that includes step/path selection functions and different behavioral and demographic states.

While habitat preferences might change according to availability (Mysterud & Ims, 1998), or other factors such as density (van Beest, McLoughlin, Mysterud, & Brook, 2016), various studies have shown the importance of habitat as a refuge to minimize risk (Atwood et al., 2004; Bouyer et al., 2015; Broekhuis et al., 2013). Therefore, if we wish to conserve subdominant carnivore species, such as cheetahs, in an area with high densities of predators and competitors, including humans, future planning of new wildlife areas has to consider habitats that can provide a refuge.

In conclusion, we strongly believe that habitat selection studies, using a multiscale approach, of species under human pressure are important, as they can predict which areas are essential for conservation.

## CONFLICT OF INTEREST

None declared.

## AUTHORS’ CONTRIBUTIONS

BK and FB conceived the ideas, collected, processed and analyzed the data, interpreted the results and wrote the manuscript. BK mapped the human footprint and FB deployed/removed the collars and created the habitat map.

## DATA ACCESSIBILITY

Data points with their extracted values for the three anthropogenic and five environmental variables for the six scales are available from the Dryad Digital Repository: https://doi.org/10.5061/dryad.8t0m6v7.
