# Supplementary material for: Living on the edge: Multiscale habitat selection by cheetahs in a human‐wildlife landscape
Source: Ecol Evol. 2018 Jul 9;8(15):7611–23. doi: 10.1002/ece3.4269 (PMC6106172; doi:10.1002/ece3.4269)

## Supporting Information

**Table S1** Overview of cheetahs collared within the study area. Dates are presented in dd/mm/yyyy. The number of data points include both the used points (GPS locations from the collars) and available points (randomly generated points at a 1:1 ratio) for each individual.

| Cheetah ID | Gender | Begin date of data collection | End date of data collection | Total no. of days | No. of data points |
|---|---|---|---|---|---|
| F01 | Female | 17/09/2015 | 15/01/2016 | 120 | 1168 |
| F02 | Female | 11/04/2015 | 10/06/2015 | 60 | 404 |
| M01 | Male | 08/03/2017 | 16/08/2017 | 162 | 1204 |
| M02 | Male | 19/10/2016 | 16/08/2017 | 301 | 2328 |
| M03 | Male | 23/02/2017 | 16/08/2017 | 174 | 1289 |
| M04 | Male | 17/10/2016 | 11/02/2017 | 117 | 868 |

**Table S2** Summary of the different habitat types found in the study area. Habitat classification was based on three different habitat structures: open, semi-closed and closed.

| **Habitat structure** | **Habitat type** | **Description** |
|---|---|---|
| **Open** | Bare ground | Areas with no vegetation i.e. rocky outcrops, eroded areas, roads etc. |
| | Grassland | Open grasslands with the occasional tree or clump of bushes. Grass species incl. Red oat grass (*Themeda triandra*), Thatch grass (*Hyparrhenia rufa*) and Sweet pitted grass (*Bothriochloa insculpa*) and tree species incl. Vachellia sp., Balanites |
| **Semi-closed** | Mixed scrub | Vachellia and different bushes |
| | Bushes | Orange-leaved croton (*Croton dichogamus*), Ol Kinyei (*Eulclea divinorum*) |
| | Whistling thorn | Whistling thorn (*Vachellia drepanolobium*) |
| | Vachellia woodland | Incl various Vachellia sp. Often open understory with tall trees |
| **Closed** | Dense | Dense woodland vegetation. Species incl. *Warburgia ugandensis*, *Vachellia xanthophloea*, *Euclea divinorum* and *Tarchonanthus camphoratus.* |

**Table S3** The different habitats found in the study area and the wildlife areas. Open habitat and semi-closed habitat are the most dominant types of habitat found in both areas. No data represents areas with cloud coverage on the satellite picture, making it impossible to determine the habitat type in that area.

| Habitat type | Study area ($km^2$) | Study area % | Wildlife areas ($km^2$) | Wildlife areas % |
|---|---|---|---|---|
| **No data** | 8.2 | 0.1 | 0.03 | 0.0 |
| **River** | 62.4 | 1.1 | 27.6 | 1.1 |
| **Open habitat** | 2558.5 | 44.4 | 1716.6 | 66.0 |
| **Closed habitat** | 376.4 | 6.5 | 89.2 | 3.4 |
| **Semi-closed habitat** | 2303.0 | 40.0 | 758.4 | 29.2 |
| **Agriculture** | 453.4 | 7.9 | 9.5 | 0.4 |
| **Total** | 5761.9 | 100 | 2601.3 | 100 |

**Table S4** The models used in the univariate analysis to select for the most important variable per class (bold).

| Class | Variable | AICc | $\Delta_i$ |
|---|---|---|---|
| Anthropogenic pressure | **Human footprint (1440m)** | **9633.9** | **0.00** |
| | Distance to main roads | 9895.1 | 261.27 |
| Habitat type | **Semi-closed habitat (90m)** | **9831.7** | **0.00** |
| | Open habitat (90m) | 9913.1 | 81.34 |
| Habitat structure | **Edge density (180m)** | **9715.6** | **0.00** |
| | Patch density (90m) | 9865.9 | 150.37 |

**Fig. S1** The relative probability of cheetah use within the study area at night and during the day determined by human footprint density, wildlife areas, semi-closed habitat, edge density, and slope. The fitted lines are presented with the 95% confidence intervals in grey.

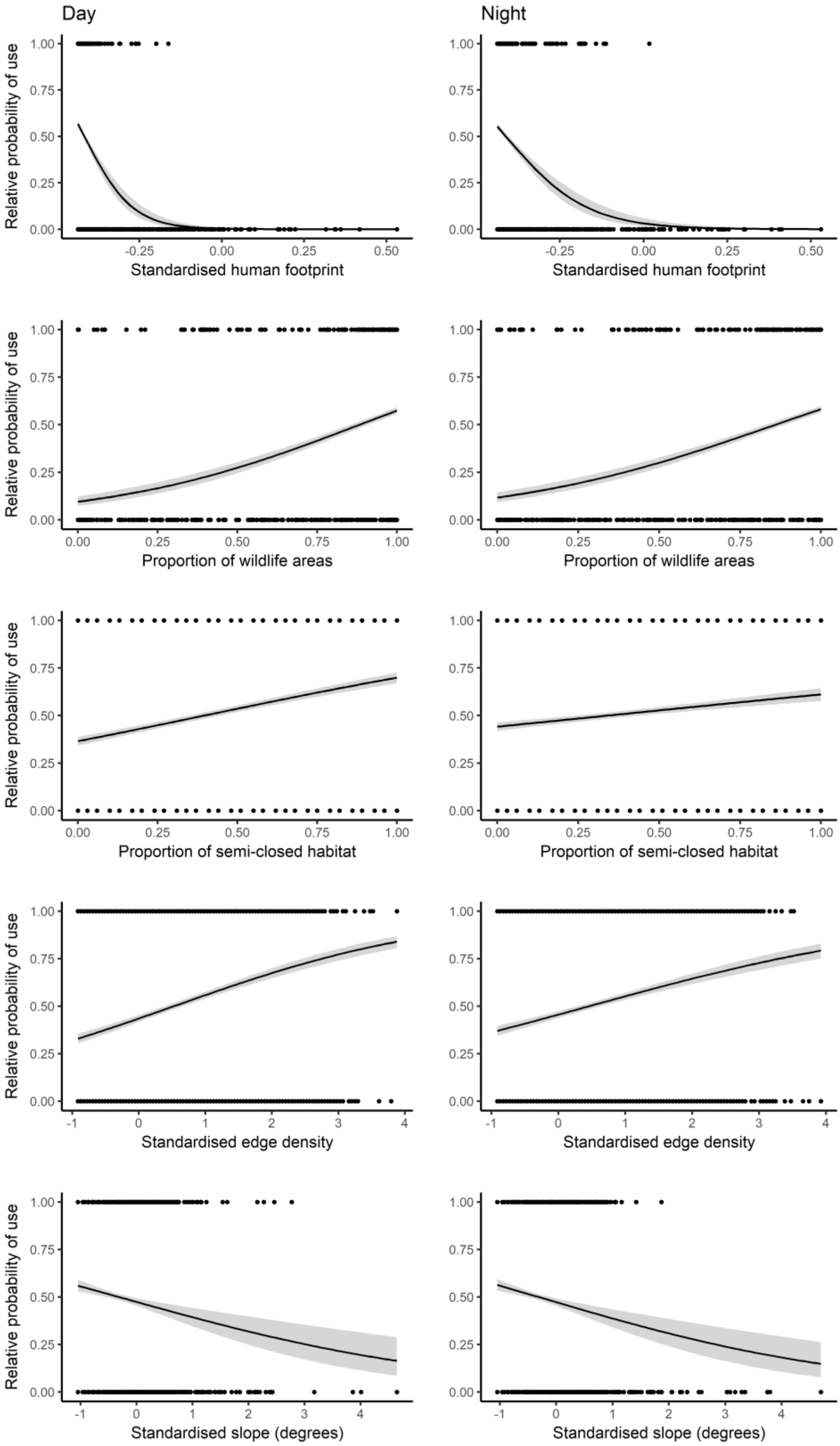

**Fig. S2** Map with all the collected data points of the six cheetahs collared in the Maasai Mara, Kenya with the wildlife areas projected in white.

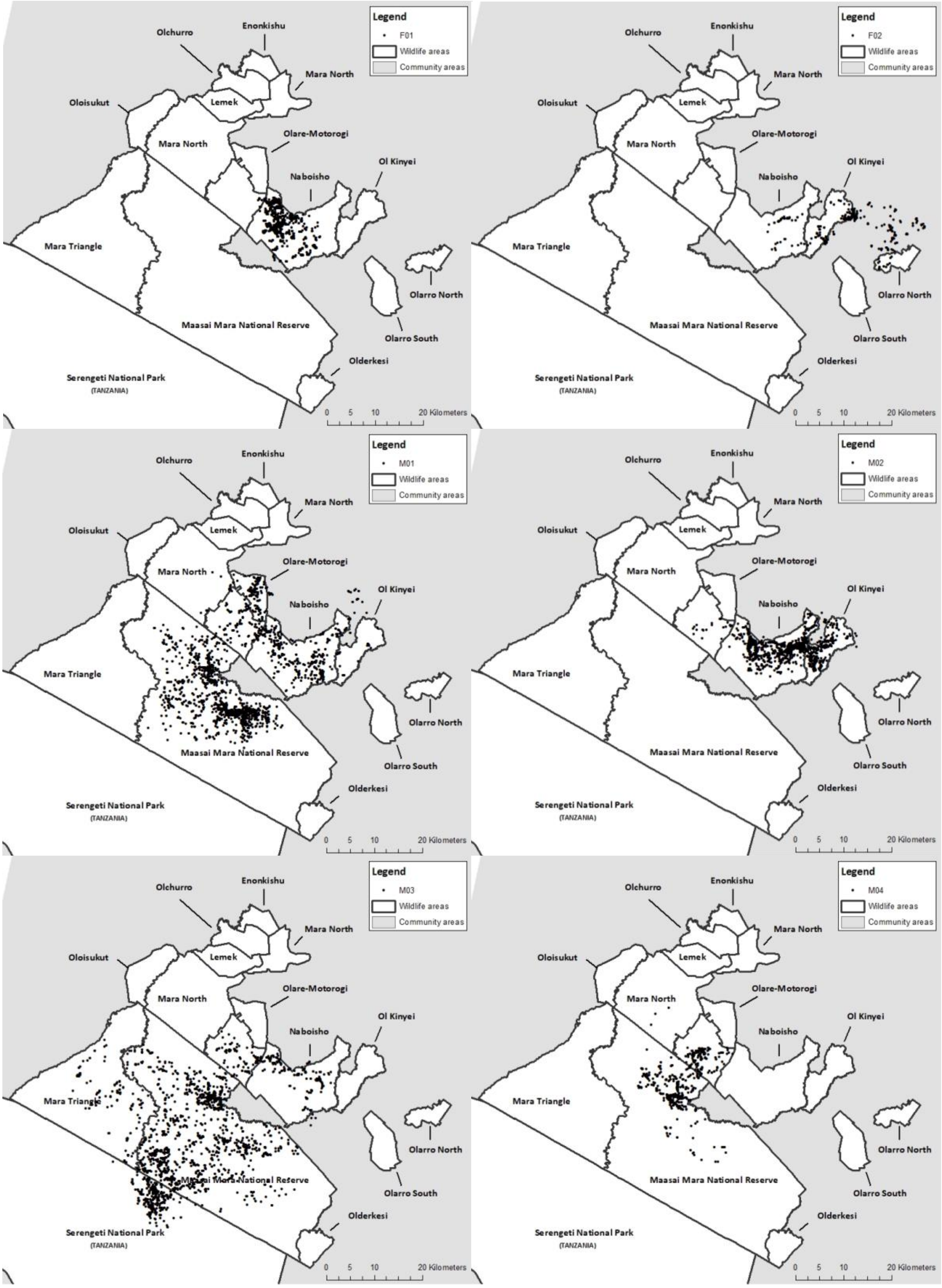

Supplement: Supplementary file 1 [file ECE3-8-7611-s001.pdf]
